# Marital status and gambling disorder: a longitudinal study based on national registry data

**DOI:** 10.1186/s12888-023-04697-w

**Published:** 2023-03-28

**Authors:** André Syvertsen, Tony Leino, Ståle Pallesen, Otto R. F. Smith, Børge Sivertsen, Mark D. Griffiths, Rune Aune Mentzoni

**Affiliations:** 1 Department of Psychosocial Science, University of Bergen, P.O. Box 7807, Bergen, 5020 Norway; 2 Norwegian Competence Center for Gambling and Gaming Research, University of Bergen, Bergen, Norway; 3 Deparment of Health Promotion, Norwegian Institute of Public Health, Bergen, Norway; 4 Department of Teacher Education, NLA University College, Bergen, Norway; 5 Department of Research & Innovation, Helse Fonna HF, Haugesund, Norway; 6 International Gaming Research Unit, Psychology Department, Nottingham Trent University, Nottingham, UK

**Keywords:** Risk factors, Gambling disorder, Registry data, Relationship status, Marriage, Divorce

## Abstract

**Background:**

Marital status is a robust correlate of disordered gambling, but few studies have examined the direction of this association.

**Methods:**

The present study used a case–control design by including all adults receiving their first gambling disorder (GD) diagnosis between January 2008 to December 2018 (Norwegian Patient Registry, *n* = 5,121) and compared them against age and gender matched individuals with other somatic/psychiatric illnesses (Norwegian Patient Registry, *n* = 27,826) and a random sample from the general population (FD-Trygd database, *n* = 26,695). The study examined marital status before GD, getting divorced as a risk factor for future GD, and becoming married as a protective factor of future GD.

**Results:**

The findings indicated an 8–9 percentage points higher prevalence of unmarried people and about a 5 percentage points higher prevalence of separation/divorce among those that subsequently experienced GD compared to controls. Logistic regressions showed that transition through divorce was associated with higher odds of future GD compared to illness controls (odds ratio [OR] = 2.89, *95% CI* [2.41, 3.45]) and the general population (OR = 2.83 [2.36, 3.38]). Logistic regressions also showed that transition through marriage was associated with lower odds of future GD compared to illness controls (OR = 0.62, *CI* [0.55, 0.70]) and the general population (OR = 0.57, *CI* [0.50, 0.64]).

**Conclusions:**

Social bonds have previously been shown to impact physical and mental health, and the findings of the study emphasize the importance of considering social network history and previous relationship dissolution among individuals with GD.

## Background

Marital status is a robust correlate of disordered gambling, a form of gambling characterized by lack of control and harm caused to the gambler and others [1, 44]. Individuals with disordered gambling are more likely to be single and to be divorced [1, 5]. Still, few studies have investigated the directionality between marital status and disordered gambling. Divorce could happen because of gambling problems but it is also possible that divorce could predispose disordered gambling. Relatedly, marriage may protect against subsequent disordered gambling. Answering questions regarding directionality requires longitudinal studies. This may ultimately help decide how associations between marital status and disordered gambling should inform therapy and prevention efforts.

Previous reviews on marital status and disordered gambling have indicated marital status a risk factor for disordered gambling while being agnostic regarding its directionality (e.g., [1, 20]. Risk factors can be defined as measurable characterizations of individuals within a specified population that can be used to divide the population into low-risk and high-risk groups [25]. Risk factors can be viewed as either fixed (e.g., ethnicity, genotype) or variable (e.g., age, marital status). Variable risk factors must precede the outcome of interest (here disordered gambling) rather than only appearing concomitantly or as a consequence, often termed as correlates [25]. Protective factors may be understood likewise but inversely (i.e., the protective factor results in reduced risk for the future outcome) [22].

Associations between disordered gambling and relationship difficulties are often understood causally, implying that gambling problems lead to relationship difficulties and break-ups [21]. This appears a plausible route given that studies have consistently found individuals to report distress due another individual’s gambling [10]. Disordered gambling in one individual is estimated to affect six others on average, among whom spouses/partners report the most distress [12, 13, 41]. This includes a wide range of psychological and emotional difficulties, alongside higher prevalence of divorce [19]. Untruthfulness, and possible illegal behaviors such as stealing money from one’s spouse may particularly damage relationships [30]. Family dysfunction such as impaired communication, emotional responsiveness and familial problem-solving, increases along with severity of gambling problems [5]. Relatedly, relationship satisfaction is inversely associated with severity of gambling problems [16].

The association between marital status and disordered gambling may also be understood in different ways. Being unmarried or divorced may potentially predispose an individual for excessive gambling. In the case of being unmarried, less social and financial obligations may increase the risk of excessive gambling as the individual experiences fewer relationship incitements for reducing gambling involvement. Having unmarried status would also preclude potential positive effects associated with marriage, such as social control and support (see below). Lack of social support has been associated with more severe gambling problems [34]. Excessive gambling may also develop or be exacerbated as a way of coping with relationship break-ups (i.e., seeing marital status change as a transitional event). Relationship dissolution has been associated with increased risk for psychopathology and poorer physical health (including early death) [42, 51]. Divorce typically represents a psychologically stressful life event and gambling motivated by emotional coping has shown to predict disordered gambling in this regard [7, 32].

Less attention has been given to marriage as a potential protective factor for disordered gambling. Research generally suggests that being married is associated with better physical and mental health [18, 24, 27]. Marriage has also been associated with reduced risk of alcohol use disorder which has been termed the ‘marriage effect’ [23, 28]. Marriage may be protective in that spouses often monitor and control each other’s alcohol drinking and/or marriage may instill a general expectation for the individual to control their alcohol drinking behavior (i.e., to *“shape up”*) [23, 49]. Such social control involves intentionally trying to influence another’s behavior and can be positive (e.g., using positive reinforcement, modeling) or negative (e.g., pressuring, restricting), with positive control being shown to promote health behaviors, and increase well-being and relationship satisfaction [11]. It is conceivable a similar ‘marriage effect’ might be observed for gambling such as spouses intentionally encouraging alternative activities to gambling for example (positive social control). Marriage might also instill an expectation to restrict gambling involvement to prioritize financial and social obligations that follow marriage.

It is not known whether gender moderates the relationship between marital status and GD. More broadly, there are also conflicting findings as to whether gender can moderate the relationship between marital status and psychological health. Leopold [29] investigated gender differences in outcomes following divorce over a 32-year period and found that men reported reduced well-being short-term compared to women, while women experienced stronger reduction in income long-term compared to men. However, other studies have not found gender differences in post-divorce and post-marriage trajectories (e.g., [47, 48, 52].

Previous studies on marital status and disordered gambling have mostly utilized general population samples and screening measures for disordered gambling, often collapsing groups that display moderate gambling risk and problem gambling in order to achieve sufficient statistical power [1]. Gambling disorder (GD) represents a more severe category and a diagnosis marked by persistent and recurring pattern of disordered gambling that is associated with substantial distress or impairment [35]. Studies on marital status that include participants with GD are typically small, with participants recruited through convenience sampling [5].

In view of these methodological limitations, the present study aimed to examine exposure to divorce as risk factor for subsequent GD diagnosis as well as exposure to marriage as a protective factor against subsequent GD diagnosis. Variable risk/protective factors require that the factor precedes the outcome. Therefore, the present study focused on marital status before GD diagnosis and transitional events in marital status happening before GD diagnosis. The latter involves either transitioning from marriage to separation/divorce or from being unmarried to marriage. Moreover, the present study explored gender differences relating to these longitudinal associations. The study overcomes limitations in previous research by use of registry-based data covering a period of 11 years allowing for high powered analyses of nationwide data concerning GD. The association between marital status and general ill-health [42, 51] was also be accounted for. More specifically, this case–control study compared those receiving their first GD diagnosis to individuals that have previously received a somatic or psychiatric diagnosis, as well as individuals from the general population. The study was guided by the following three research questions (RQs):

1. Are individuals who subsequently receive their first gambling disorder diagnosis more likely to be unmarried or separated/divorced compared to those that do not receive a gambling disorder diagnosis?
2. Is going through a divorce associated with increased odds of experiencing a gambling disorder diagnosis compared to remaining married? If so, is this association moderated by gender?
3. Is getting married associated with reduced odds of experiencing gambling disorder diagnosis compared to remaining unmarried? If so, is this association moderated by gender?

## Methods

### Participants and procedure

The study comprised a population-based case–control study of all individuals in Norway 18 years or older receiving their first GD diagnosis within specialist health services between January 2008 to December 2018 (*n* = 5,121). Information on participants and controls was collected from the Norwegian Patient Registry (NPR), providing information about diagnosis and time for diagnosis, and the FD-Trygd database providing information about dates for change in marital status. Data from the two registries were linked using unique 11-digit National identity numbers. NPR contains health information on patients in Norwegian specialist health services and has included unique national birth numbers necessary for linking registry information since 2008 [4]. FD-Trygd contains demographic information, including marital status, as well as information on work status and social benefits for the Norwegian population from 1992 and onwards [46]. Participants with GD were frequency matched on age and gender characteristics using two control/contrast populations of 30,000 randomly drawn individuals with illness diagnoses other than GD (from NPR) and from the general population (from FD-Trygd), aiming for approximately five matched controls per GD case. After removing duplicate cases and cases missing marital status information, the following sample sizes were obtained: NPR illness controls (*n* = 27,826) and FD-Trygd general controls (*n* = 26,695).

The study received ethical approval from the Regional Committee for Medical and Health Related Research Ethics in Western Norway (no. 30393) and the Norwegian Centre for Research Data. The approval included a waiver of informed consent because the data was anonymized before the authors got access to it. The ethical approval covers the stated aims of the current study. The study was conducted in accordance with the Helsinki Declaration. A Data Protection Impact Assessment (DPIA) was also made in collaboration with the University of Bergen and approved by the Institute of Psychosocial Science, University of Bergen.

### Measures

Demographic information included age, gender, and marital status. Age and gender information was extracted based on information in the National identity number which is assigned at birth or at permanent migration into Norway. Information about marital status was collected from FD-Trygd and was categorized into unmarried, married (including registered partnership), separated/divorced, and widowed. Changes in marital status within study period January 2008 to December 2018 were recorded and summarized into four categories: No change in marital status (0), one change in marital status (1), two changes in marital status (2), and three or more changes in marital status (3). Information was not available on cohabitation status and ethnicity. Gambling disorder was defined according to medical diagnosis in NPR which is based on the ICD-10 code F63.0 for pathological gambling [53].

### Statistical analysis

All statistical analyses were conducted with R version 4.1.1. Descriptive statistics and statistics to inform RQ1 included distribution of age at baseline, gender, marital status at baseline, and number of changes in marital status within the study period (i.e., an indication of marital status variability across the study period), stratified by case and control groups. RQ2 and RQ3 were examined by two pairs of logistic regressions, one pair for examining divorce as a risk factor for GD and another pair for examining marriage as a protective factor for GD. Each pair included a logistic regression against illness control participants from NPR and a logistic regression against general population control participants from FD-Trygd. Unconditional logistic regression analyses were used to examine if exposure was associated with the odds of receiving a GD diagnosis, with the matching variables age and gender included as control variables, and with an interaction term between gender and marital status to investigate if the associations were dependent upon gender. A two-step approach was used by separately estimating a main effect only model and a model including an interaction term for gender. Unconditional logistic regressions have been shown to be suitable for analysis in case–control designs that frequency match participants on demographic variables such as age and gender (i.e., studies that employ *“loose matching”*) [26, 33]. Both adjusted and unadjusted odds ratios are presented (i.e., the effect of each predictor when controlling for other predictors versus not controlling for other predictors).

When examining the association between exposure to divorce and the odds of receiving a GD diagnosis, the study included participants who were married at baseline and defined those that got divorced during the study period as exposed. Participants who subsequently got re-married or became widowed during the study period were excluded from this analysis to examine divorce only. For the GD sample, only changes in marital status before diagnosis were included. The study also censored any marital changes within the control groups that happened after the median time to GD diagnosis (72 months for this analytic sample) to allow for comparable follow-up periods.

When examining the association between exposure to marriage and odds of receiving GD diagnosis, the study included participants who were unmarried at baseline and defined those that married during study period as exposed. Participants who subsequently got separated/divorced after marriage were excluded from this analysis to examine marriage only. For the GD sample, only changes in marital status before diagnosis were included. The study also censored any marital changes within the control groups that happened after the median time to GD diagnosis (85 months for this analytic sample) to allow for comparable follow-up periods.

## Results

Descriptive data are provided in Table 1 broken down by case and control group. Chi-square test on marital status categories was significant, informed RQ1 and shows that individuals who eventually received a GD diagnosis were more likely to be unmarried or separated/divorced at baseline compared to controls, and less likely to be married at baseline compared to controls (χ^2^ [df = 6, *n* = 59,642] = 487.50, *p* < 0.001). Moreover, individuals who subsequently received a GD diagnosis were more likely to experience multiple changes in marital status throughout the study period (χ^2^ [df = 6, *n* = 59,642] = 129.61, *p* < 0.001).

**Table 1:** Participant characteristics at baseline

Sample	GD (*n* = 5,121)	Illness control (*n* = 27,826)	General control *(n* = 26,695)	*p*-value^1^
Women	935 (18.3%)	5,038 (18.1%)	5,193 (19.5%)	< 0.001
Age in 2008				< 0.001
Median (IQR)	29 (22, 39)	29 (22, 39)	30 (22, 39)	
Mean (SD)	30.9 (12)	30.8 (12)	31.3 (12)	
Marital status at baseline				< 0.001
Unmarried	3,674 (71.7%)	17,828 (64.1%)	16,819 (63.0%)	
Married	914 (18.9%)	8,404 (30.2%)	8,345 (31.3%)	
Separated/divorced	510 (10.0%)	1,510 (5.4%)	1,444 (5.4%)	
Widowed	23 (0.4%)	84 (0.3%)	87 (0.3%)	
Marital status changes^2^				< 0.001
0	4,024 (78.6%)	22,324 (80.2%)	21,123 (79.1%)	
1	812 (15.9%)	4,730 (17.0%)	4,757 (17.8%)	
2	224 (4.4%)	633 (2.3%)	685 (2.6%)	
3 +	61 (1.2%)	139 (0.5%)	130 (0.5%)	

*Note*. ^1^Pearson’s Chi-squared test for categorical; One-way ANOVA for continuous. ^2^During study period January 2008 to December 2018. Total percentage slightly exceeds 100 in some cases due to rounding

Logistic regression results on analysis of exposure to divorce on GD are provided in Table 2 and informed RQ2. The interaction terms between gender and exposure were not statistically significant (NPR control: OR = 1.16, 95% CI [0.76, 1.75]; FD-Trygd control: OR = 1.21, 95% CI [0.79, 1.82]), so only main effect analyses are reported in the table. ORs were similar for the adjusted and unadjusted analysis. The analytic samples were comparable in terms of age distributions: *M* = 50 (10) among GD cases, *M* = 50 (10) among NPR controls, and *M* = 51 (10) among FD-Trygd controls. Distribution of gender differed somewhat, with the proportion of women being lower among cases with GD (22%) compared to NPR controls (27%) and FD-Trygd controls (28%). The results showed that getting divorced was associated with a higher odds ratio of receiving a GD diagnosis. The strength of association was comparable using both types of control groups. Using individuals with other illnesses as controls, those getting divorced had 2.89 (95% CI [2.41, 3.45]) times the odds of getting a GD diagnosis compared to individuals who remained married during the exposure period, based on the adjusted analysis. Using individuals from the general population as controls, those getting divorced had 2.83 (95% CI [2.36, 3.38]) times the odds of getting a GD diagnosis compared to individuals who remained married during the exposure period, based on the adjusted analysis.

**Table 2:** Logistic regressions for divorce on odds for first gambling disorder diagnosis

	Against NPR illness control (*n* = 7,441)			Against FD-Trygd general control (*n* = 7,443)		
Predictor	OR^1^	95% CI^1^	*p*-value	OR^1^	95% CI^1^	*p*-value
**Unadjusted analysis**						
Age in 2008	1.00	[1.00, 1.01]	0.261	1.00	[0.99, 1.00]	0.403
Gender						
Men (reference)	—	—		—	—	
Women	0.77	[0.65, 0.90]	0.002	0.73	[0.62, 0.86]	< 0.001
Exposure						
Married (reference)	—	—		—	—	
Divorce	2.82	[2.36, 3.37]	< 0.001	2.82	[2.36, 3.37]	< 0.001
**Adjusted analysis**						
Age in 2008	1.01	[1.00, 1.02]	0.025	1.00	[0.99, 1.01]	0.720
Gender						
Men (reference)	1.00	—		1.00	—	
Women	0.75	[0.64, 0.89]	< 0.001	0.73	[0.61, 0.86]	< 0.001
Exposure						
Married (reference)	1.00	—		1.00	—	
Divorce	2.89	[2.41, 3.45]	< 0.001	2.83	[2.36, 3.38]	< 0.001

*Note*. ^1^*OR* odds ratio, *CI* confidence interval. GD cases = 885

Logistic regression results on analysis of exposure to marriage on GD are provided in Table 3 and informed RQ3. The interaction terms between gender and exposure were not statistically significant (NPR control: OR = 0.91, 95% CI [0.64, 1.27]; FD-trygd control: OR = 0.80, 95% CI [0.56, 1.11]), therefore, only main effect analyses are reported in the table. ORs were similar between the adjusted and unadjusted analysis (although the effect of gender was statistically significant in the unadjusted analysis but not in the adjusted analysis). The analytic samples were comparable in terms of age and gender distributions. For age: *M* = 37 years (*SD* = 9) among GD cases, *M* = 36 years (*SD* = 9) among NPR controls, and *M* = 36 years (*SD* = 9) among FD-Trygd controls. For the proportion of women: GD (14%), NPR controls (13%) and FD-Trygd controls (14%). The results showed that getting married was associated with a lower odds ratio of getting GD diagnosis, and the strength of association was comparable using both the respective types of control groups. Using individuals with other illnesses as controls, those getting married had 0.62 (95% CI [0.55, 0.70]) times the odds of getting a GD diagnosis compared to individuals who remained unmarried during the exposure period, based on the adjusted analysis. Using individuals from the general population as controls, those getting married had 0.57 (95% CI [0.50, 0.64]) times the odds of getting a GD diagnosis compared to individuals who remained unmarried during the exposure period, based on the adjusted analysis.

**Table 3:** Logistic regressions for marriage on odds for first gambling disorder diagnosis

Predictor	Against NPR illness control (*n* = 16,925)			Against FD-Trygd general control (*n* = 15,940)		
	OR^a^	95% CI^a^	*p*-value	OR^a^	95% CI^a^	*p*-value
**Unadjusted analysis**						
Age in 2008	1.01	[1.00, 1.01]	< 0.001	1.01	[1.00, 1.01]	0.006
Gender						
Men (reference)	1.00	—		1.00	—	
Women	1.12	[1.01, 1.24]	0.034	1.00	[0.90, 1.11]	0.985
Exposure						
Unmarried (reference)	1.00	—		1.00	—	
Marriage	0.64	[0.56, 0.72]	< 0.001	0.58	[0.51, 0.66]	< 0.001
**Adjusted analysis**						
Age in 2008	1.01	[1.00, 1.01]	< 0.001	1.01	[1.00, 1.01]	< 0.001
Gender						
Men (reference)	1.00	—		1.00	—	
Women	1.10	[0.99, 1.22]	0.076	1.00	[0.90, 1.11]	0.976
Exposure						
Unmarried (reference)	1.00	—		1.00	—	
Marriage	0.62	[0.55, 0.70]	< 0.001	0.57	[0.50, 0.64]	< 0.001

^a^*OR* Odds ratio, *CI* Confidence interval. GD cases = 3,610

## Discussion

The present study examined marital status as a risk/protective factor for subsequent first GD diagnosis. One of the study aims was to examine if individuals that went on to receive their first GD diagnosis were more likely to be unmarried or separated/divorced compared to control populations at baseline (RQ1). The results showed that within the GD population there was an 8–9 percentage points higher prevalence of unmarried individuals compared to controls (case: 72% vs. controls: 64% illness and 63% general population). Further, prevalence of separation/divorce were nearly twice as high at baseline among those that would go on to receive GD compared to controls (case: 10% vs. controls 5.4% for the respective control groups). These results suggest that those who receive a diagnosis of GD represent a group of individuals with reduced social networks and who experience more relationship dissolution compared to individuals with other forms of ill-health or from the general population. Marital status represents a structural indication of an individual’s social connectedness and experiencing social connection through a spouse is beneficial for both physical and mental well-being [17, 40]. Relatedly, lack of social support has been associated with more severe gambling problems and poorer prognosis in treatment [34]. It has also been found that loneliness can mediate a positive association between being unmarried/divorced/widowed and having disordered gambling for men [8]. The results also showed that individuals with GD had more variability in marital status across the study period compared to the control groups. Although differences between study groups were of small magnitude, they were still statistically significant, due to the present study’s large sample size.

The study examined how changes in marital status affected the odds for GD diagnosis (RQ2 and RQ3). The results showed that going through a divorce was associated with 2.89 and 2.83 higher odds of receiving a subsequent GD diagnosis in the case group compared to the NPR illness group and FD-Trygd general population group, respectively. It is notable that the increased odds for GD diagnosis was similar when using a general population control group and a control group based on individuals with different types of psychiatric and somatic diagnoses. The similar ORs for receiving GD diagnosis when using both types of control groups suggest similar number of divorces across the analyzed period for married individuals in both types of control groups. This appears somewhat surprising because there is a frequently observed association between divorce and mental/somatic ill-health, although this might stem from divorce influencing health rather than ill-health influencing divorce [42, 51]. The illness control group in the present study only included individuals with existing diagnoses so differences in number of divorces due to ill-health influencing divorce would be captured primarily. If divorce influences health rather than the reverse, then this could explain why rates of divorce were similar between the illness control group and general control group.

Most individuals who go through a divorce experience little or no negative long-term effects (i.e., > 12 months) which raises the question as to what predicts poor outcomes such as physical illness and psychopathology (including disordered gambling) following divorce [3]. Sbarra et al. [43] propose that excessive rumination, lack of a clear self-concept (i.e., individuals not knowing who they are as a person following divorce), and poor sleep may predispose poor outcomes following divorce. These factors could then promote long-term stress which might predispose some individuals’ excessive gambling because they use gambling as a coping strategy. Gambling as a means of regulating affective states is a well-established pathway in the development of disordered gambling [2, 6, 14].

Getting married was in the present study found to be associated with 0.62 and 0.57 lower odds of receiving a subsequent GD diagnosis in the case group compared to the NPR illness group and FD-Trygd general population group, respectively. This suggests a protective effect of marriage and the effect was similar when using a general population control group and when using a control group based on individuals with different types of psychiatric and somatic diagnoses. Marriage has been associated with reduced risk of developing alcohol use disorder [23, 28]. Kendler et al. [23] proposed that a ‘marriage effect’ related to alcohol use disorder was primarily due to social control between spouses (i.e., couples monitoring and controlling each other’s alcohol drinking). This is likely also the case with gambling (i.e., couples monitoring and controlling each other’s gambling behavior). Marriage can also confer social support which is related to better health behaviors and less stress [50], also potentially protecting against disordered gambling behavior.

Finally, the study examined if gender moderated the relationship between transition in marital status and odds for being diagnosed with GD (the conditional aspect of RQ2 and RQ3). The results showed non-significant findings for both types of marital transitions. For divorce, this contrasts with previous research suggesting that divorce has stronger impact on men’s short-term wellbeing and women’s long-term income level, compared to the other gender [29]. However, a recent Danish study [47] did not find any gender-related differences in post-divorce trajectories between men and women. The authors argued that such gender differences were less likely to appear in egalitarian societies, such as the Danish. Norway in this regard is like Denmark and this could explain a similar pattern in results. For marriage, the lack of gender differences in the present study matched that of previous studies [48, 52].

### Strengths and limitations

The present study employed data from national registries data, which have several benefits. Data collection is done automatically and without being intrusive, which eliminates the risk for recall bias, social desirability bias, and research demand characteristics. Previously, very few studies have investigated marital status in relation to the more severe GD category and sample sizes have been relatively low in these studies (e.g., [5]. Using registry data allows researchers to get access to much higher numbers of participants which leads to high statistical power. In the present study, this allowed for investigating the more specific research questions that required sub-groups related to marital status (e.g., those starting as married and then getting divorced). Notably, having time-specific data on both marital status and GD diagnosis made it possible to investigate directionality which previous studies have not been able to do.

Some study limitations should be noted. The present study only included treatment-seeking individuals with GD diagnosis and did not include individuals with less severe problem gambling. It is estimated that only between 5–20% of those with problem gambling seek treatment [31]. Individuals with gambling problems who seek treatment tend to report more severe relationship difficulties compared to those who do not seek treatment [37]. The present study also only included age and gender as control variables. Disordered gambling has also been shown to associated with other correlates such as ethnicity, socio-economic status, and poor physical and mental health [1]. Individuals with various somatic and psychiatric diagnosis where such correlates are relative frequent were included as a control group and results were comparable between two control groups which strengthens the generalizability of the study’s findings. Still, it cannot be ruled out that these other variables (e.g., ethnicity, socio-economic status) could have impacted the results as explanatory or confounding variables.

Another limitation concerns the age and gender matching process used. More specifically, individuals with GD were matched on age and gender in the total sample, but not within specific subgroups analyzed such as those transitioning from marriage to divorce. Age distribution was comparable between cases and controls in this subgroup but there were some differences in gender distribution between case and control groups. Results were comparable when assessing GD cases against both control groups despite these groups showing some variation in gender distribution. There might also be unidentified confounding factors that can explain the associations observed in the present study. In the case of divorce for example, individuals may alternatively first develop excessive gambling, facilitating break-up/divorce, which then motivates the individual to seek treatment (and receive a GD diagnosis).

Finally, information on cohabitation status was not available in the present study. This means that it was not possible to account for potential increased or reduced risk for GD diagnosis among individuals starting cohabiting during the study period (analogue to marriage) or break-up from cohabiting during the study period (analogue to divorce). Not accounting for cohabitation could have led to reduced effect sizes for transitions through marriage or divorce regarding odds/risks for GD diagnosis. For instance, in the analysis concerning individuals starting out unmarried (Table 3), some individuals might have already been cohabiting or started cohabiting later which would mean that any potential reduced risk for future GD diagnosis associated with cohabitation would be attributed to the “unmarried” reference group. In Norway, cohabitation has been shown to be associated with increases in well-being that are almost identical to the increases associated with marriage [45]. Individuals who are cohabiting with children have previously been found to have comparable reduction in risk for alcohol use disorder as married individuals [23]. Therefore, future research should examine if the same relationship between marriage/divorce and risk for GD diagnosis also holds for cohabitation/break-up from cohabitation.

### Implications and conclusions

In the present study, individuals that subsequently received their first GD diagnosis were more likely to show indications of reduced social connectedness (i.e., more likely to be unmarried and separated/divorced). Moreover, it was found that transitioning through divorce or marriage was associated with increased or reduced odds of GD diagnosis, respectively. Notably, examining marital status/changes before GD diagnosis demonstrated that these factors are risk or protective factors for developing GD. Future studies may expand on the findings reported here by examining the relationship between marital quality and disordered gambling. Marital quality has been found to be positively associated with physical and mental health [36, 38]. Additionally, studies have also suggested that divorce can lead to improved health in cases where there was low marital quality [9, 15].

It is not possible to conclude that transitions in marital status causally affect the development of GD based on the present study’s design. However, this might be the case as a large body of previous research substantiates the association between relationship dissolution and poorer physical and mental health [42, 51], as well as between social connectedness and better physical and mental health [17, 40].

Treatment for GD include efforts to minimize harms caused by disordered gambling on current relationships [12, 39]. The present study’s findings emphasize the importance of considering both individuals’ previous and current social factors, including social network history and experiences with relationship dissolution. Interventions that increase an individual’s level and quality of social connectedness might in turn improve their GD therapy prognosis as well as overall wellbeing [34, 40].

## Data Availability

This study used data from national registries and is available upon application only. The data is not publicly available due to restrictions from the Norwegian Patient Registry and the FD-Trygd registry.

## Abbreviations

GD: Gambling disorder
NPR: Norwegian Patient Registry
FD-Trygd: Forløpsdatabasen-Trygd
