# Correction: Marital status and gambling disorder: a longitudinal study based on national registry data

**DOI:** 10.1186/s12888-024-05519-3

**Published:** 2024-02-19

**Authors:** André Syvertsen, Tony Leino, Ståle Pallesen, Otto R. F. Smith, Børge Sivertsen, Mark D. Grifths, Rune Aune Mentzoni

**Affiliations:** 1 Department of Psychosocial Science, University of Bergen, P.O. Box 7807, 5020 Bergen, Norway; 2 Norwegian Competence Center for Gambling and Gaming Research, University of Bergen, Bergen, Norway; 3 Department of Health Promotion, Norwegian Institute of Public Health, Bergen, Norway; 4 Department of Teacher Education, NLA University College, Bergen, Norway; 5 Department of Research & Innovation, Helse Fonna HF, Haugesund, Norway; 6 International Gaming Research Unit, Psychology Department, Nottingham Trent University, Nottingham, UK

**Correction: BMC Psychiatry 23: 199 (2023)**

**https://doi.org/10.1186/s12888-023-04697-w**

Following the publication of the original article [1], multiple errors were identified in the sections and Tables 1 and 2. The correct tables are given below and the changes in the abstract, results and discussion sections have been highlighted in **bold typeface**.

The incorrect Table 1 is:
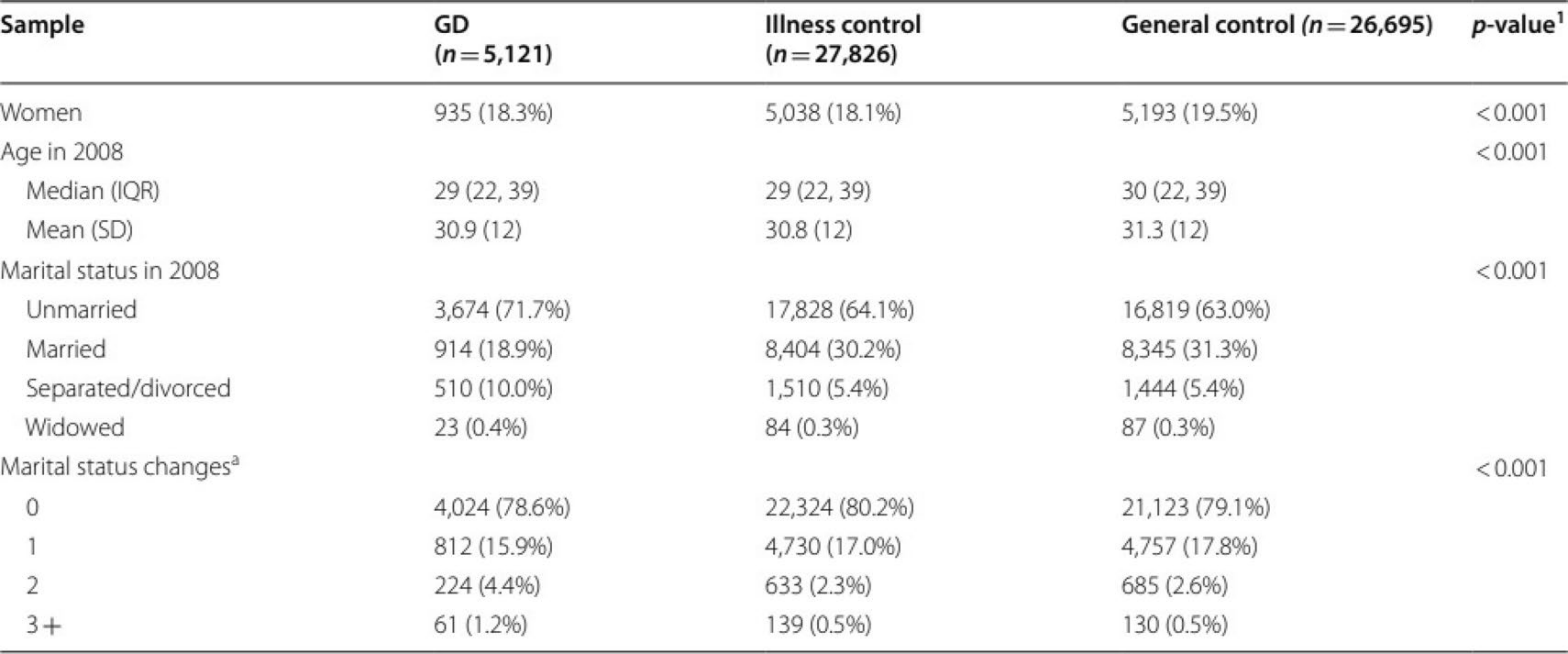


The correct Table 1 is:

**Table 1:** Participant characteristics at baseline

Sample	GD (*n* = 5,121)	Illness control (*n* = 27,826)	General control *(n* = 26,695)	*p*-value^1^
Women	935 (18.3%)	5,038 (18.1%)	5,193 (19.5%)	< 0.001
Age in 2008				< 0.001
Median (IQR)	29 (22, 39)	29 (22, 39)	30 (22, 39)	
Mean (SD)	30.9 (12)	30.8 (12)	31.3 (12)	
Marital status at baseline				< 0.001
Unmarried	3,674 (71.7%)	17,828 (64.1%)	16,819 (63.0%)	
Married	914 (18.9%)	8,404 (30.2%)	8,345 (31.3%)	
Separated/divorced	510 (10.0%)	1,510 (5.4%)	1,444 (5.4%)	
Widowed	23 (0.4%)	84 (0.3%)	87 (0.3%)	
Marital status changes^2^				< 0.001
0	4,024 (78.6%)	22,324 (80.2%)	21,123 (79.1%)	
1	812 (15.9%)	4,730 (17.0%)	4,757 (17.8%)	
2	224 (4.4%)	633 (2.3%)	685 (2.6%)	
3 +	61 (1.2%)	139 (0.5%)	130 (0.5%)	

*Note*. ^1^Pearson's Chi-squared test for categorical; One-way ANOVA for continuous. ^2^During study period January 2008 to December 2018. Total percentage slightly exceeds 100 in some cases due to rounding

The incorrect Table 2 is:
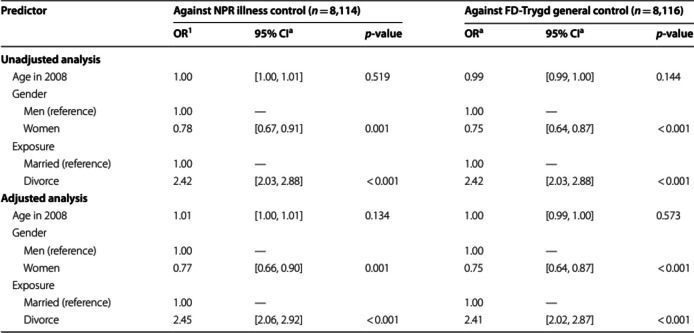


The correct Table 2 is:

**Table 2:** Logistic regressions for divorce on odds for first gambling disorder diagnosis

	Against NPR illness control (*n* = 7,441)			Against FD-Trygd general control (*n* = 7,443)		
Predictor	OR^1^	95% CI^1^	*p*-value	OR^1^	95% CI^1^	*p*-value
**Unadjusted analysis**						
Age in 2008	1.00	[1.00, 1.01]	0.261	1.00	[0.99, 1.00]	0.403
Gender						
Men (reference)	—	—		—	—	
Women	0.77	[0.65, 0.90]	0.002	0.73	[0.62, 0.86]	< 0.001
Exposure						
Married (reference)	—	—		—	—	
Divorce	2.82	[2.36, 3.37]	< 0.001	2.82	[2.36, 3.37]	< 0.001
**Adjusted analysis**						
Age in 2008	1.01	[1.00, 1.02]	0.025	1.00	[0.99, 1.01]	0.720
Gender						
Men (reference)	1.00	—		1.00	—	
Women	0.75	[0.64, 0.89]	< 0.001	0.73	[0.61, 0.86]	< 0.001
Exposure						
Married (reference)	1.00	—		1.00	—	
Divorce	2.89	[2.41, 3.45]	< 0.001	2.83	[2.36, 3.38]	< 0.001

*Note*. ^1^*OR* odds ratio, *CI* confidence interval. GD cases = 885

Abstract-Results

The sentence currently reads: Logistic regressions showed that transition through divorce was associated with higher odds of future GD compared to illness controls (odds ratio [OR] = 2.45, 95% CI [2.06, 2.92]) and the general population (OR = 2.41 [2.02, 2.87]).

The sentence should read: Logistic regressions showed that transition through divorce was associated with higher odds of future GD compared to illness controls (odds ratio [OR] = **2.89**, *95% CI* [**2.41, 3.45**]) and the general population (OR = **2.83** [**2.36, 3.38**]).

Results

The incorrect paragraph is: Logistic regression results on analysis of exposure to divorce on GD are provided in Table 2 and informed RQ2. The interaction terms between gender and exposure were not statistically significant (NPR control: OR = 1.11, 95% CI [0.74, 1.66]; FD-Trygd control: OR = 1.15, 95% CI [0.76, 1.72]), so only main effect analyses are reported in the table. ORs were similar between the adjusted and unadjusted analysis. The analytic samples were comparable in terms of age distributions: M = 50 (9) among GD cases, M = 50 (10) among NPR controls, and M = 51 (10) among FD-Trygd controls. Distribution gender differed somewhat, with the proportion of women being lower among cases with GD (23%) compared to NPR controls (26%) and FD-Trygd controls (28%). The results showed that getting divorced was associated with a higher odds ratio of receiving a GD diagnosis. The strength of association was comparable using both types of control groups. Using individuals with other illnesses as controls, those getting divorced had 2.45 (95% CI [2.06, 2.92]) times the odds of getting a GD diagnosis compared to individuals who remained married during the exposure period, based on the adjusted analysis. Using individuals from the general population as controls, those getting divorced had 2.41 (95% CI [2.02, 2.87]) times the odds of getting a GD diagnosis compared to individuals who remained married during the exposure period, based on the adjusted analysis.

The correct paragraph is: Logistic regression results on analysis of exposure to divorce on GD are provided in Table 2 and informed RQ2. The interaction terms between gender and exposure were not statistically significant (NPR control: OR = **1.16, 95% CI [0.76, 1.75**]; FD-Trygd control: OR = **1.21**, 95% CI [**0.79, 1.82**]), so only main effect analyses are reported in the table. ORs were similar for the adjusted and unadjusted analysis. The analytic samples were comparable in terms of age distributions: M = 50 (**10**) among GD cases, M = 50 (10) among NPR controls, and M = 51 (10) among FD-Trygd controls. Distribution of gender differed somewhat, with the proportion of women being lower among cases with GD (**22%**) compared to NPR controls (**27%**) and FD-Trygd controls (28%). The results showed that getting divorced was associated with a higher odds ratio of receiving a GD diagnosis. The strength of association was comparable using both types of control groups. Using individuals with other illnesses as controls, those getting divorced had **2.89** (95% CI [**2.41, 3.45**]) times the odds of getting a GD diagnosis compared to individuals who remained married during the exposure period, based on the adjusted analysis. Using individuals from the general population as controls, those getting divorced had 2.83 (95% CI [**2.36, 3.38**]) times the odds of getting a GD diagnosis compared to individuals who remained married during the exposure period, based on the adjusted analysis.

Discussion

The incorrect sentence is: The results showed that going through a divorce was associated with 2.45 and 2.41 higher odds of receiving a subsequent GD diagnosis in the case group compared to the NPR illness group and FD-Trygd general population group, respectively.

The correct sentence is: The results showed that going through a divorce was associated with **2.89** and **2.83** higher odds of receiving a subsequent GD diagnosis in the case group compared to the NPR illness group and FD-Trygd general population group, respectively.

The original article [1] has been corrected.
